# The Microbiome and Antibiotic Resistome in Soil under Biodegradable Composite Carbon Source Amendment

**DOI:** 10.3390/jox13030027

**Published:** 2023-08-15

**Authors:** Zhongchen Yang, Yanhong Lou, Xianghui Yan, Hong Pan, Hui Wang, Quangang Yang, Yajie Sun, Yuping Zhuge

**Affiliations:** National Engineering Research Center for Efficient Utilization of Soil and Fertilizer Resources, College of Resources and Environment, Shandong Agricultural University, Tai’an 271018, China; zcyang@sdau.edu.cn (Z.Y.); yanhonglou@sdau.edu.cn (Y.L.); yanxh@sdau.edu.cn (X.Y.); hongpan@sdau.edu.cn (H.P.); huiwang@sdau.edu.cn (H.W.); sttzzy@sdau.edu.cn (Q.Y.); sunyajie@sdau.edu.cn (Y.S.)

**Keywords:** biodegradable microplastics, composite carbon source, antibiotic resistance genes, metagenomic sequencing, microbial community

## Abstract

The decomposition of biodegradable composite carbon sources generates a large amount of biodegradable microplastics, which may not only furnish microbial denitrification, but might also pose potential environmental risks. In the present study, the effects of different dosages of a biodegradable composite carbon source on the microbial communities, the nitrogen metabolic pathways and the antibiotic resistome were explored through Illumina MiSeq sequencing analysis and metagenomic analysis. The results of partial least-square discriminant analysis (PLS-DA) and analysis of similarity (ANOSIM) demonstrated that the response of the bacterial community to a biodegradable composite carbon source was more obvious than the fungal community. The application of biodegradable microplastics diminished the complexity of the microbial communities to some extent and obviously stimulated denitrification. Antibiotics resistance gene (ARG) dispersal was not evidently accelerated after the addition of biodegradable composite carbon source. *Lysobacter*, *Methylobacillus*, *Phyllobacterium*, *Sinorhizobium*, *Sphingomonas* from *Proteobacteria* and *Actinomadura*, *Agromyces*, *Gaiella* and *Micromonospora* from *Actinobacteria* were the major ARG hosts. Overall, the addition of a biodegradable composite carbon source shaped microbial communities and their antibiotic resistance profiles in this study.

## 1. Introduction

Synthetic and inorganic fertilizers are widely used in agriculture due to their low costs and significant effect in increasing crop yields. Thanks to the application of nitrogen fertilizers, the global crop production has increased by approximately half [1]. However, it is not the case that the higher the amount of nitrogen fertilizers used, the higher the crop yields obtained. The overuse of nitrogen fertilizers reduces nitrogen use efficiency and is not beneficial to increasing crop yields. Considering the nitrogen use efficiency of crops ranging from 30% to 50%, more than half of a nitrogen fertilizer has not effectively applied to crops, but has dissipated through leaching, volatilization, and microbial utilization [2]. The remaining nitrogen fertilizer in soil would be converted to ammonia, ammonium and nitrate, which might pose a threat to human health and the ecological environment after excessive accumulation [3]. Hence, an effective approach for excessive nitrate removal from soil is urgently needed to protect public health and terrestrial ecosystems.

Bioremediation is a kind of remediation technology decomposing hazardous substances or transforming them to substances with less or non-toxic forms through the metabolic activities of microorganisms [4]. The performance of bioremediation is impacted by contaminant characteristics, pH, redox potential, nutrients, moisture, and temperature [5,6]. Normally, intrinsic microorganisms are able to achieve bioremediation of most pollutants, that is, intrinsic bioremediation. To accelerate microbial growth and reinforce bioremediation, some specific substrates are supplemented to serve as electron donors, nutrients and pH buffers [4]. In our previous study, the application of a composite carbon source (<2 mm), made of pretreated corncob and biodegradable polybutylene succinate, successfully strengthened the bioremediation performance of excessive nitrate in soil under atrazine stress [7]. However, limited information was available about the effects of a composite carbon source on microbial community diversity, nitrogen removal pathways, and the potential risks on the environment.

Biodegradable composite carbon sources, which can be readily decomposed by microorganisms, have become a popular alternative to non-degradable plastics, since biodegradable composite carbon sources can not only provide habitats for microorganisms, but also serve as an exogenous carbon source to supply microbial metabolism [8]. Nowadays, a biodegradable composite carbon source has been applied to the advanced treatment of low-carbon–nitrogen ratio sewage [9,10], and has raised high hopes among researchers. The microbial decomposition of a biodegradable composite carbon source will generate a large amount of plastic debris, of which particle sizes of less than 5 mm are known as microplastics [11]. At present, the spread of ARGs has become one of the most challenging environmental issues [12,13]. Moreover, microplastics act as a reservoir for ARGs and contribute to the spread of ARGs [14]. Even worse, pesticides are recognized to be associated with the enrichment of ARGs through cross-resistance and shared plasmids [15,16]. Unfortunately, few studies have extensively addressed whether biodegradable microplastics exacerbate the enrichment of ARGs caused by pesticides.

Therefore, the objectives of this study were: (1) to characterize the composition and diversity of microbial communities in treatments with different dosages of composite carbon source; (2) to elucidate the effects of a composite carbon source on the interactions between bacterial and fungal communities; (3) to reveal the changes in nitrogen cycling pathways and involved functional genes; and (4) to clarify the distribution of the antibiotic resistome, potential risks and possible ARG hosts. These findings would provide insights into the control of the ARG spread from the application of biodegradable plastics.

## 2. Materials and Methods

### 2.1. Materials and Sample Collection

In our previous study, five kinds of composite carbon sources were prepared using polybutylene succinate and corncob with different pretreatment methods, of which the composite carbon source with the best biodegradable performance was used in this study. In detail, corncob was soaked in NaOH solution (0.01 mol/L) to remove ingredients difficult to biodegrade, washed with deionized water and dried to constant weight. After, equal weights of polybutylene succinate and pretreated corncob were added into the internal mixer, blended smoothly together and heated to prepare the composite carbon source [17]. The composite carbon source was crushed and passed through a 2 mm sieve. Analytical-grade atrazine (purity > 98%) was dissolved in methanol for further use.

Soil samples were collected from a wheat–corn rotation field with long-term application of organic fertilizer. This area possesses a typical temperate and monsoonal climate, with an annual average temperature of 12.9 °C and an annual average precipitation of 659.0 mm. The collected soil was immediately transported to the laboratory on ice, sieved (2 mm), and air dried.

### 2.2. Microcosm Experiment

To systematically decipher the microbial compositions, functions and antibiotic resistomes, the microcosm experiment was performed. Before the formal experiment, the soil moisture content was adjusted to 60% of the maximum field water-holding capacity, and the soil samples were preincubated at 25 °C in the dark for 1 week to activate microbiota. The recommended dose of atrazine (3 mg/kg) was selected to simulate the practical application of pesticide [18]. In detail, 500 g of the soil sample was evenly stirred when spraying the methanol solution of atrazine (3 mg/mL). Subsequently, 4500 g of the soil sample was added and thoroughly homogenized to acquire soil with a final concentration of 3 mg/kg. After being left for 2 h in the fume cupboard to achieve complete evaporation of methanol, every 50 g of the soil sample, a composite carbon source (0, 5 and 25 g/kg, namely C0, C5 and C25) and KNO_3_ solution (1.5 mg/mL NO_3_^−^-N) was successively fed into 250 mL serum bottle. Treatments without a composite carbon source (C0) served as controls. In order to remove the oxygen, sufficient N_2_ was injected into the serum bottles, which were afterwards sealed with rubber plugs. The soil water content was maintained at 60% of the maximum field water-holding capacity by periodic addition of sterile water. Each treatment was performed in triplicate. All of the serum bottles were placed in an incubator (GXZ-436, Ningbo Jiangnan Instrument Factory, Ningbo, China) at 25 °C in the dark. A destructive sampling strategy was performed on the 42nd day [7].

### 2.3. DNA Extraction and Illumina MiSeq Sequencing Analysis

The structure of the soil microbial community was investigated using Illumina MiSeq sequencing. Approximately 0.30 g of the soil sample was used to extract the total microbial genomic DNA using a microbial DNA extraction kit (Biocolors, Shanghai, China). The quantity of the extracted DNA was determined using a NanoDrop-2000 spectrophotometer (Nanodrop Technologies, Wilmington, NC, USA) and its quality was visualized by 1% agarose gel electrophoresis. The V3–V4 region of the bacterial 16S rRNA genes and the ITS1 region of the fungal rRNA genes were amplified using the primers 338F/806R and ITS1F/ITS2R, respectively. Triplicate PCR reactions were performed in a total volume of 20 μL, containing 4 μL of 5x FastPfu buffer, 2 μL of 2.5 mM dNTPs, 0.8 μL of each primer, 0.4 μL of FastPfu DNA Polymerase, 0.2 μL of BSA solution, 10 ng of template DNA, and ddH_2_O to make up a total volume of 20 μL. The PCR thermal cycling conditions were as follows: initial denaturation at 95 °C for 3 min followed by either 35 cycles (for fungi) or 27 cycles (for bacteria) of denaturing at 95 °C for 30 s, annealing at 55 °C for 30 s, extension at 72 °C for 45 s, single extension at 72 °C for 10 min, and end at 10 °C. The PCR products were purified using a 2% agarose gel and a DNA purification kit (Tiangen, Beijing, China).

Illumina MiSeq sequencing of the bacterial 16S rRNA and fungal ITS rRNA genes was conducted using the Illumina MiSeq PE 300 platform (Illumina, San Diego, CA, USA) at Majorbio Bio-pharm Technology Co., Ltd., Shanghai, China. Data analysis was conducted using the Mothur software (http://www.mothur.org, accessed on 30 March 2023). Sequences that were shorter than 50 base pairs (bp) with a quality score below 20 were removed from the data sets. The remaining sequences were clustered into operational taxonomic units (OTUs) with a similarity threshold value of 97%.

### 2.4. Metagenomic Analysis

Metagenomic analysis was conducted to investigate the gene metabolic functions to clarify the hosts, mobile antibiotic resistome and potential mechanism of the ARGs. The extracted DNA was fragmented to construct paired-end sequencing, and sequencing analysis was performed using Illumina NovaSeq 6000 (Illumina Inc., San Diego, CA, USA). To ensure the quality of subsequent analyses, reads with a length less than 50 bp, an average quality value less than 20 or containing an N-base were removed using fastp (https://github.com/OpenGene/fastp, accessed on 10 April 2023). Then, the data were assembled using the Multiple Megahit strategy, and contigs higher than 300 bp were retained to conduct open reading frame (ORF) prediction using Prodigal [19]. The predicted genes were clustered using CD-HIT (http://www.bioinformatics.org/cd-hit/, accessed on 10 April 2023), and genes with maximal sequences were picked out to construct a non-redundant gene catalog.

For taxonomic annotations, the predicted genes were annotated against the integrated Non-Redundant Protein Sequence Database using BLASTP (http://blast.ncbi.nlm.nih.gov/Blast.cgi, accessed on 10 April 2023) with an e-value of 10^−5^. The annotations of Kyoto Encyclopedia of Genes and Genomes (KEGG) were carried out by BLASTP with an e-value of 10^−5^. Based on the results of metagenomic KEGG annotation, the abundance of key functional genes in different nitrogen cycle pathways was screened, including organic nitrogen metabolism (*gltB*, *gltD*, *gdhA*, *gdhA*, *glnB*, *ureA*, *ureB* and *ureC*), denitrification (*nirS*, *nirK*, *norB*, *norC*, *nosZ*, *narG*, *narH*, *narI*, *nxrA*, *nxrB*, *napA*, *napB* and *napC*), nitrogen transport (*NRT*, *nrtA*, *nrtB*, *nrtC* and *nrtD*), dissimilatory nitrate reduction to ammonium (*nrfA*, *nrfH*, *nirB* and *nirD*), assimilatory nitrate reduction (*nasA*, *nasB*, *narB* and *nirA*), nitrification (*pmoA-amoA*, *pmoB-amoB*, *pmoC-amoC* and *hao*) and nitrogen fixation (*nifD*, *nifH* and *nifK*).

To identify the types, subtypes and resistance mechanism of ARGs, the predicted ORFs were identified as ARG-like ORFs by searching nucleotide sequences using DeepARG (https://bitbucket.org/gusphdproj/deeparg-ss/src/master/, accessed on 1 June 2023), which was a merged database (http://bench.cs.vt.edu/ftp/deeparg/scripts/, accessed on 1 June 2023) containing the Antibiotic Resistance Genes Database (ARDB), the Comprehensive Antibiotic Resistance Database (CARD), and the Universal Protein Resource (UNIPROT) database [20]. The abundance of ARG-like reads was normalized by the metagenomic data set after quality control [21]. Mobile genetic elements (MGEs) were identified using DIAMOND (https://github.com/bbuchfink/diamond accessed on 1 June 2023) against the MGEs90 database (https://bench.cs.vt.edu/ftp/data/databases/MGEs90.fasta, accessed on 1 June 2023).

### 2.5. Network Analysis

The microbial co-occurrence analysis has great advantages in revealing deeper microbial associations and exploring the responses of microbial functions to environmental changes beyond simple richness and composition [22,23]. In this study, the co-occurrence networks were constructed to show the potential relationships between bacterial and fungal genera and to explore the interactive relationships among ARGs and their hosts based on the Spearman rank correlation coefficient. All the nodes in the network have strong positive or negative relationships (absolute value of r > 0.8) with statistical significance (*p* < 0.05). Gephi software (version 0.9.2) was used to visualize the analysis results.

### 2.6. Statistical Analyses

Differences between groups regarding the relative abundance of dominated phyla and nitrogen cycling pathways were analyzed by one-way ANOVA (*p* < 0.05) using SPSS 26.0 software (IBM, Chicago, IL, USA). The results were considered to be statistically significant when *p* < 0.05. Partial least-square discriminant analysis (PLS-DA) and analysis of similarity (ANOSIM) were performed to assess the differences in the bacterial and fungal communities among the different treatments. The significant relationships between bacterial and fungal genera and the interactive relationships among ARGs and hosts were visualized in co-occurrence networks.

## 3. Results and Discussion

### 3.1. The Effect of a Composite Carbon Source on the Microbial Community

**Figure 1:**
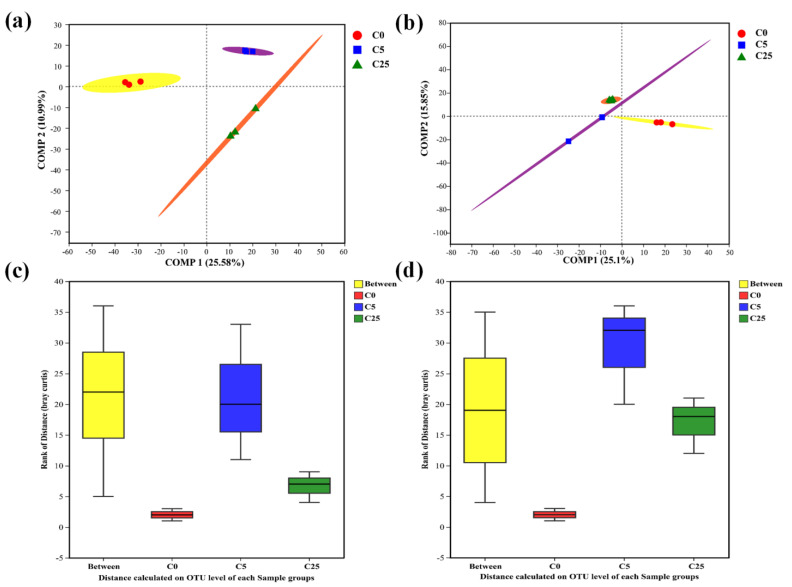
PLS−DA and ANOSIM of bacterial (**a**,**c**) and fungal communities (**b**,**d**), respectively, in treatments of C0 (soil without composite carbon source), C5 (soil with 5 g/kg composite carbon source) and C25 (soil with 25 g/kg composite carbon source). Each treatment has three replicates.

A total of 352,863 and 395,002 high-quality sequences were obtained for the soil bacterial and fungal communities, respectively. Coverage values ranging from 0.9793 to 0.9999 indicated sufficient sequencing depth and accuracy. The PLS-DA analysis showed that both bacterial and fungal communities in groups with a composite carbon source (C5 and C25) were well separated from the control group (C0) along the COMP1 axis (Figure 1a,b). ANOSIM was used to test the null hypothesis that there were no constituent differences between different groups. The R statistics from ANOSIM of bacterial and fungal communities were 0.6296 and 0.1770, respectively (*p* < 0.05) (Figure 1c,d). It was concluded that bacterial communities of all groups could be classified as a separation with R > 0.5, while the fungal communities of the soil samples could hardly be separated with R < 0.25 [24]. The results of PLS-DA analysis and ANOSIM demonstrated that the effect of a composite carbon source on the bacterial community was more significant than that on the fungal community.

**Figure 2:**
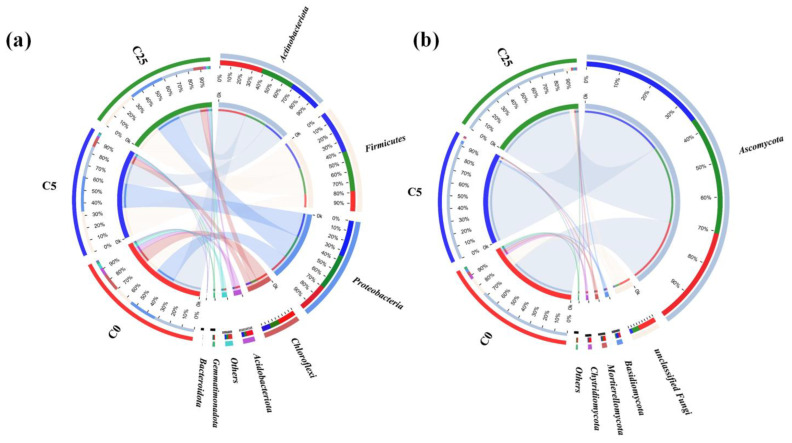
The compositions of bacterial (**a**) and fungal communities (**b**) at the phylum level in treatments of C0 (soil without composite carbon source), C5 (soil with 5 g/kg composite carbon source) and C25 (soil with 25 g/kg composite carbon source). Each treatment has three replicates.

The predominant bacterial phyla (relative abundance > 1.00%) in the soil samples included *Actinobacteriota*, *Firmicutes*, *Proteobacteria*, *Chloroflexi*, *Acidobacteriota*, *Gemmatimonadota* and *Bacteroidota* (Figure 2a), accounting for over 95% of the total bacterial community. C0 harbored higher *Actinobacteriota* (33.49%), *Chloroflexi* (14.55%), *Acidobacteriota* (6.42%), *Gemmatimonadota* (1.75%) and *Bacteroidota* (1.13%) than C5 and C25, while the relative abundance of *Firmicutes* and *Proteobacteria* was lower. Compared to bacterial communities, there were only four phyla with a relative abundance higher than 1.00%, namely *Ascomycetes*, *Basidiomycetes*, *Mortierelleomycota* and *Chytridomycetes* (Figure 2b). *Ascomycetes* achieved obviously higher relative abundance in C5 (93.51%) and C25 (89.46%) than in C0 (74.28%) (*p* < 0.05), while the relative abundance of *Mortierelleomycota* and *Chytridomycetes* decreased from 2.92% and 3.37% to 1.07–1.58% and 0.56–0.86%, respectively.

*Actinobacteriota*, *Proteobacteria*, *Chloroflexi* and *Acidobacteria* have been recognized as the major phyla of bacteria in soil [25,26], which is in accordance with this study. *Actinobacteriota* has the ability to complete the decomposition of organic carbon [27]. *Acidobacteriota* is competent at degrading polysaccharides of plant and fungal origin, and contributes to soil ecosystems and the carbon cycle [28]. *Chloroflexi* is a type of anaerobic bacteria widely observed in aquatic and terrestrial environments which can be involved in fermentation of organic compounds [29]. *Gemmatimonadota* has been found to have the ability to degrade organic matter [30]. *Bacteroidota* can degrade complex organic matters, such as polysaccharides and proteins [31], and favors higher values of soil pH [32]. It was reported that the change in environmental factors might affect *Bacteroidetes* more than the fertilizer [33]. *Firmicutes* and *Proteobacteria* are dominated by heterotrophic species [34]. Many of the bacteria in *Proteobacteria* can participate in the nitrogen cycle and readily grow in nutrient-rich soil [35,36]. The decomposition of a composite carbon source can generate a large number of small molecular organic carbon, which facilitated the growth of heterotrophic bacteria and increased the relative abundance of Firmicutes and Proteobacteria. As for fungi, *Ascomycota* and *Basidiomycota* are the two dominant phyla of fungi in global soils [37]. Ascomycota contributes to the decomposition process and the nutrient cycle [38], and the distinctly higher relative abundance of *Ascomycota* in C5 and C25 might be induced by abundant lignocellulose in the composite carbon source. Basidiomycetes have the capacity to degrade lignocellulose and xenobiotic compounds [39].

**Figure 3:**
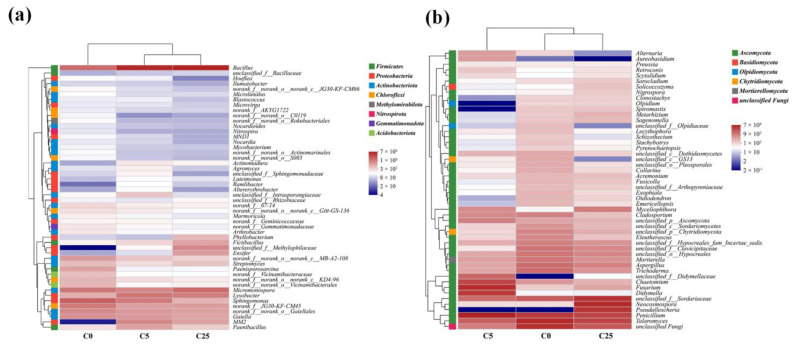
The compositions of bacterial (**a**) and fungal communities (**b**) at the genus level in treatments of C0 (soil without composite carbon source), C5 (soil with 5 g/kg composite carbon source) and C25 (soil with 25 g/kg composite carbon source). Each treatment has three replicates.

The top 50 bacterial genera are presented in Figure 3a. *Bacillus* was the most dominant genus in all the soil samples, and acquired a higher relative abundance in C5 (26.73%) and C25 (24.60%) than in C0 (7.85%). In addition, the relative abundance of *Lysobacter* and *Paenibacillus* in C5 and C25 was also higher than in C0. *Bacilli* plays an important role in organic fermentation in anoxic soil conditions [40], and the remarkably higher relative abundance in C5 (26.73%) and C25 might be due to the application of the composite carbon source. As a genus widely found in soils, some members of *Lysobacter* can increase the disease suppression of soil phytopathogens and act as efficient antagonists of phytopathogens [41]. *Paenibacillus* is generally associated with the plant roots, which promote plant growth and can be exploited in agriculture [42]. With respect to fungal genera (Figure 3b), the highest relative abundance of *Talaromyces* and *Aspergillus* was observed in C0, *Neocosmospora* and *Pseudallescheria* tended to be concentrated in C25, while *Penicillium*, *Fusarium*, *Didymella* and *Chaetomium* were inclined to aggregate in C0. Talaromyces is an excellent decomposer of organic matter and actively participates in biogeochemical cycles [43]. *Fusarium* and *Neocosmospora* might contribute the decomposition of soil organic matter and N_2_O production [44]. Known as a cellulolytic fungus, *Chaetomium* possesses the potential ability to degrade cellulosic waste [45]. *Penicillium* can withstand high levels of atrazine, utilize atrazine as a nitrogen source and degrade it [46]. *Fusarium* and *Aspergillus* may be able to degrade atrazine via N-dealkylation [47]. In consistent incubation conditions, the dosage of a composite carbon source might play an extremely important role in the shaping of fungal communities.

### 3.2. Network Analysis of the Microbial Community

**Figure 4:**
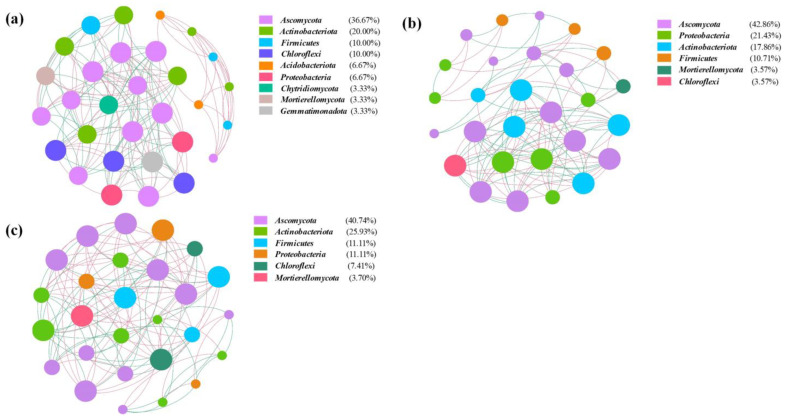
The co-occurrence network of microbial communities in C0 (**a**), C5 (**b**), and C25 (**c**) at the genus level. The nodes of genera are colored by phylum, and their sizes are proportional to the number of connections. The negative and positive correlations are represented by red and green connections, respectively.

The co-occurrence network analysis was used to simplify complex interactions among bacterial and fungal microbes, identify the keystone taxa, and explore the underlying relationships among microorganisms (Figure 4). The modularity indexes in different groups were 0.612 (C0), 0.471 (C5), and 0.571 (C25), indicating that these networks had modular structures with modularity indexes higher than 0.4. The constructed networks established 284 (160 positive and 124 negative), 224 (108 positive and 116 negative), and 235 (140 positive and 95 negative) edges in C0, C5, and C25, respectively. The lower numbers of connections in C5 and C25 implied that the application of a composite carbon source diminished the complexity of the microbial communities to some extent, especially in C5. Furthermore, the C25 treatment showed more positive associations (59.57%) than C0 (56.34%) and C5 (48.21%). In the co-occurrence network, the positive associations might represent a stable network structure, with fewer competitive interactions or mutualisms in the microbial community [48]. With the increase in composite carbon source dosage, the positive associations of microbial communities decreased first and then increased. The composite carbon source can be decomposed by the degrading microorganisms in soil and generated small-molecule organic matters can be readily utilized by microorganisms [49], contributing to a highly nutritious microenvironment and significant aggregation of degrading and heterotrophic microorganisms, which alters community compositions to some extent. Therefore, compared with the nutrient-rich C25 treatment, the antagonism among microorganisms was greater in the nutrient-deficient C5 treatment.

### 3.3. Changes in Nitrogen Cycling Processes and Involved Functional Genes

**Figure 5:**
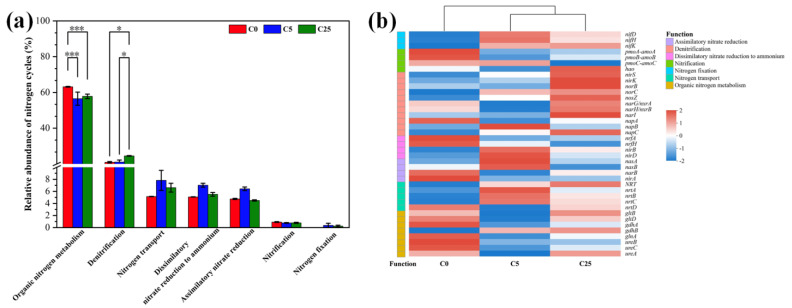
The relative abundance of nitrogen cycling processes in different treatments (**a**) and related functional genes (**b**). The symbol * and *** mean that the correlation is statistically significant at the 0.05 and 0.001 levels, respectively.

Based on the results of the KEGG annotation, the effects of a composite carbon source on nitrogen cycling processes and distribution of nitrogen functional genes were studied (Figure 5). A total of seven major nitrogen cycling processes were annotated in this study, including organic nitrogen metabolism, denitrification, nitrogen transport, dissimilatory nitrate reduction to ammonium, assimilatory nitrate reduction, nitrification and nitrogen fixation. Organic nitrogen metabolism predominated in all treatments, especially in C0 with a relative abundance of 63.17%, which was obviously higher than that of C25 (57.76%) and C5 (56.49%). The application of a composite carbon source promoted the enrichment of denitrification functional genes, especially in C25 (24.53%). The relative abundance of nitrogen transport, dissimilatory nitrate reduction to ammonium, assimilatory nitrate reduction, nitrification and nitrogen fixation was relatively low (<8%). It is notable that almost all nitrogen transport, dissimilatory nitrate reduction to ammonium, assimilatory nitrate reduction and nitrogen fixation functional genes acquired higher relative abundance in C5 and C25 than C0, which contributed to the accumulation of ammonia and corresponded to the results of our previous study [7].

In terms of the denitrification functional genes, the prevailing functional genes of denitrification in C0 and C5 were *napA* and *napB*, which can encode nitrate reductase and promote the conversion of nitrate to nitrite. In contrast, almost all of the denitrification functional genes were significantly enriched in C25, which demonstrated that the application of 25 g/kg of a composite carbon source obviously facilitated the denitrification and accumulation of crucial functional genes in C25. The decomposition of the composite carbon source generated abundant accessible organic substrates, which furnished the growth of heterotrophic microbes and impacted the composition of microbial community [50]. Ultimately, the changes in the composition of microbial communities led to variations in the composition of functional genes closely related to taxonomic composition [51].

### 3.4. The Antibiotic Resistome in the Soil Samples

**Figure 6:**
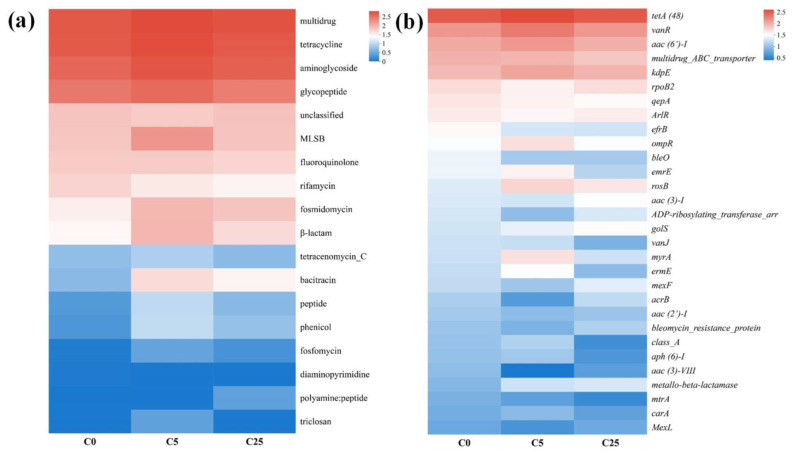
The distribution of different types (**a**) and subtypes (**b**) of ARGs in treatments of C0 (soil without composite carbon source), C5 (soil with 5 g/kg composite carbon source) and C25 (soil with 25 g/kg composite carbon source). Each treatment had three replicates.

The application of a composite carbon source did not have a significant effect on the ARG types (*p* > 0.05) (Figure 6a). The dominant ARG types in soils were multidrug, tetracycline, aminoglycoside and glycopetide resistance genes. Notably, the relative abundance of fosmidomycin, β-lactam and bacitracin resistance genes in C5 and C25 was higher than that in C0. Multidrug, aminoglycoside and β-lactam resistance genes were frequently detected in natural environments [52]. Multidrug resistance can persist in the environment for a long time because of its high solubility and chemical stability [53]. Tetracycline and β-lactam resistance gene may be derived from nature, because it appeared in the environment before clinical application [54].

A total of 126 unique ARGs subtypes were annotated in this study, and the top 30 ARG subtypes in different treatments are shown in Figure 6b, and can be clustered into 9 ARG types, including multidrug (*acrB*, *efrB*, *emrE*, *golS*, *mexF*, *MexL*, *mtrA*, *multidrug ABC transporter*, *ompR* and *rpoB2*), aminoglycoside (*aac (2’)-I*, *aac (3)-I*, *aac (3)-VIII*, *aac (6’)-I*, *aph (6)-I* and *kdpE*), glycopeptide (*bleO*, *bleomycin resistance protein*, *vanJ* and *vanR*), MLS (*carA*, *ermE* and *myrA*), β-lactam (*class A* and *metallo beta lactamase*), fluoroquinolone (*qepA*), fosmidomycin (*rosB*), rifamycin (*ADP ribosylating transferase arr*) and tetracycline (*tetA(48)*). *vanR*, *aac (6’)-I* and *tetA (48)* were predominant ARGs in all treatments. Due to long-term application of organic fertilizer, a large amount of *vanR* remained in animal manure and entered field soil, resulting in high levels of *vanR* in all the soil samples [55]. In addition, the use of organic fertilizer also led to a significant increase in the abundance of aminoglycoside gene (*aac(6′)-I*) in field soil [56]. As a kind of efflux pump, *tetA* is one of the resistance genes generally detected in soil [57] and confers cross-resistance to multiple antibiotics [58]. It was reported that *tetA (48)* can deactivate tetracycline antibiotics and serve as the dominant tetracycline resistance determinant [59]. To make matters worse, *tetA (48)* is thought to be mobile [60]. Therefore, it is necessary to assess the potential risk of ARGs transfer.

**Figure 7:**
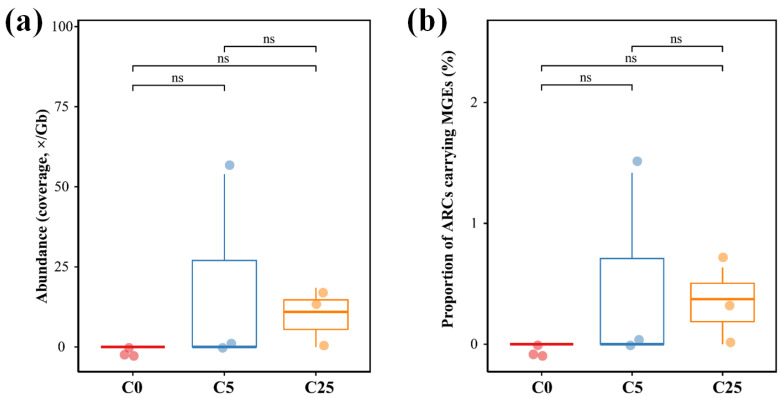
The total abundance (**a**) and proportion (**b**) of ARG-carrying MGEs in treatments of C0 (soil without composite carbon source), C5 (soil with 5 g/kg composite carbon source) and C25 (soil with 25 g/kg composite carbon source). Each treatment had three replicates.

The total abundance and proportion of ARG-carrying MGEs in the soil samples were calculated to assess differences in the potential mobility of ARGs in different treatments (Figure 7). Their co-occurrence relationship was determined when both ARGs and MGEs were located on the same contig. The largest abundance was observed in C25, with a coverage of 9.80 ×/Gb (*p* > 0.05), while no ARG-carrying MGEs were annotated in C0, similar to C5 except the soil sample showed significant deviations. The proportion of ARG-carrying MGEs in different treatments did not show significant differences (*p* < 0.05), indicating that the application of a composite carbon source did not distinctly stimulate the mobile potential of ARGs.

### 3.5. Potential Host Bacteria for ARGs

**Figure 8:**
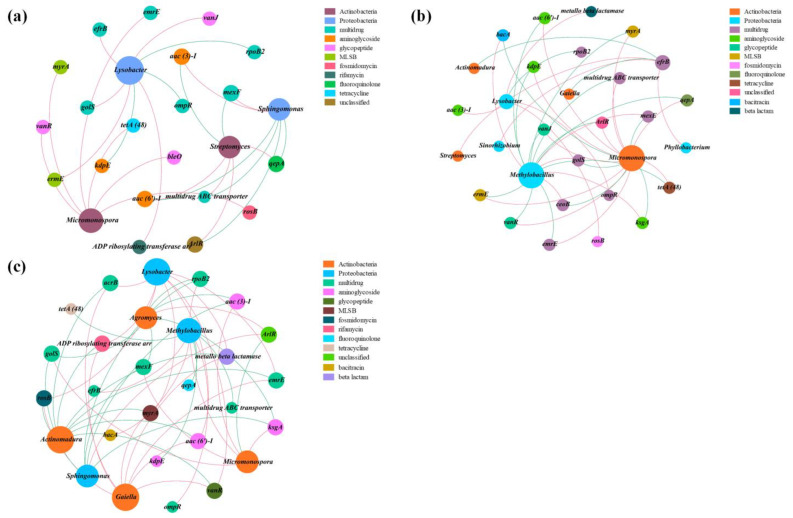
Network analysis revealing the co-occurrence patterns between ARG subtypes and bacterial genera in C0 (**a**), C5 (**b**) and C25 (**c**). The negative and positive correlations are represented by red and green connections, respectively.

The co-occurrence network of ARG subtypes and bacterial genera is depicted in Figure 8. It can be clearly seen that the correlation patterns between bacterial taxa and ARGs in C5 (42 edges) and C25 (49 edges) were much more complex than that in C0 (26 edges). Furthermore, there were more potential ARG hosts in C5 and C25, suggesting that the composite carbon source can act as a reservoir for ARGs [61], and was the dominant factor for shaping microbial communities and their antibiotic resistance profiles in this study. A total of 10 bacterial genera had significant positive correlations with ARG subtypes (*p* < 0.05), including *Lysobacter*, *Methylobacillus*, *Phyllobacterium*, *Sinorhizobium*, *Sphingomonas* from *Proteobacteria* and *Actinomadura*, *Agromyces*, *Gaiella*, *Micromonospora* and *Streptomyces* from *Actinobacteria*. In this study, *Actinobacteria* and *Proteobacteria* were the prevailing potential ARG hosts. It was reported that *Actinobacteria* and *Proteobacteria* were common ARG hosts in soil [62]. The ARGs (*aac (3)-I*, *aac (6’)-I*, *ArlR*, *efrB*, *emrE*, *golS*, *mtrA*, *rosB* and *vanR*) had positive associations with the genera from *Actinobacteria* and *Proteobacteria* and were present in all treatments, which suggested that the persistence of these ARGs was mainly regulated by the changes in *Actinobacteria* and *Proteobacteria* [63]. On the contrary, *bacA*, *ksgA*, *metallo beta lactamase* and *ompR* ARGs were only observed in C5 and C25, which might result from the co-selection of pesticide and microplastics generated from the decomposition of the composite carbon source [64,65].

## 4. Conclusions

The present study investigated the compositions of microbial communities, nitrogen transformation pathways and the antibiotic resistome under different dosages of composite carbon sources. The application of a composite carbon source had a more significant effect on the bacterial community than the fungal community, diminished the complexity of the microbial communities to some extent and promoted microbial denitrification. The application of a composite carbon source induced the selective distribution of ARG subtypes, but did not distinctly stimulate the mobile potential of ARGs. The ARG hosts mainly belonged to the *Proteobacteria* and *Actinobacteria*. These findings are important for understanding of the microbial community, function and antibiotic resistome of the biodegradable composite carbon source, and are helpful for developing risk management practices to control ARG contamination.

## Data Availability

Data are available upon request due to privacy and ethical restrictions.
